# Development of a COVID-19–Related Anti-Asian Tweet Data Set: Quantitative Study

**DOI:** 10.2196/40403

**Published:** 2023-02-28

**Authors:** Maryam Mokhberi, Ahana Biswas, Zarif Masud, Roula Kteily-Hawa, Abby Goldstein, Joseph Roy Gillis, Shebuti Rayana, Syed Ishtiaque Ahmed

**Affiliations:** 1 Department of Computer Science University of Toronto Toronto, ON Canada; 2 Indian Institute of Technology Kanpur Kanpur India; 3 Brescia University College at Western London, ON Canada; 4 Ontario Institute for Studies in Education Toronto, ON Canada; 5 Mathematics, Computer & Information Sciences State University of New York at Old Westbury Old Westbury, NY United States

**Keywords:** COVID-19, stigma, hate speech, classification, annotation, data set, Sinophobia, Twitter, BERT, pandemic, data, online, community, Asian, research, discrimination

## Abstract

**Background:**

Since the advent of the COVID-19 pandemic, individuals of Asian descent (colloquial usage prevalent in North America, where “Asian” is used to refer to people from East Asia, particularly China) have been the subject of stigma and hate speech in both offline and online communities. One of the major venues for encountering such unfair attacks is social networks, such as Twitter. As the research community seeks to understand, analyze, and implement detection techniques, high-quality data sets are becoming immensely important.

**Objective:**

In this study, we introduce a manually labeled data set of tweets containing anti-Asian stigmatizing content.

**Methods:**

We sampled over 668 million tweets posted on Twitter from January to July 2020 and used an iterative data construction approach that included 3 different stages of algorithm-driven data selection. Finally, we found volunteers who manually annotated the tweets by hand to arrive at a high-quality data set of tweets and a second, more sampled data set with higher-quality labels from multiple annotators. We presented this final high-quality Twitter data set on stigma toward Chinese people during the COVID-19 pandemic. The data set and instructions for labeling can be viewed in the Github repository. Furthermore, we implemented some state-of-the-art models to detect stigmatizing tweets to set initial benchmarks for our data set.

**Results:**

Our primary contributions are labeled data sets. Data Set v3.0 contained 11,263 tweets with primary labels (unknown/irrelevant, not-stigmatizing, stigmatizing-low, stigmatizing-medium, stigmatizing-high) and tweet subtopics (eg, wet market and eating habits, COVID-19 cases, bioweapon). Data Set v3.1 contained 4998 (44.4%) tweets randomly sampled from Data Set v3.0, where a second annotator labeled them only on the primary labels and then a third annotator resolved conflicts between the first and second annotators. To demonstrate the usefulness of our data set, preliminary experiments on the data set showed that the Bidirectional Encoder Representations from Transformers (BERT) model achieved the highest accuracy of 79% when detecting stigma on unseen data with traditional models, such as a support vector machine (SVM) performing at 73% accuracy.

**Conclusions:**

Our data set can be used as a benchmark for further qualitative and quantitative research and analysis around the issue. It first reaffirms the existence and significance of widespread discrimination and stigma toward the Asian population worldwide. Moreover, our data set and subsequent arguments should assist other researchers from various domains, including psychologists, public policy authorities, and sociologists, to analyze the complex economic, political, historical, and cultural underlying roots of anti-Asian stigmatization and hateful behaviors. A manually annotated data set is of paramount importance for developing algorithms that can be used to detect stigma or problematic text, particularly on social media. We believe this contribution will help predict and subsequently design interventions that will significantly help reduce stigma, hate, and discrimination against marginalized populations during future crises like COVID-19.

## Introduction

### Background

Individuals of Asian descent (we refer to the colloquial usage of the word prevalent in North America, where “Asian” is used to refer to people from East Asia, particularly China) have been subjected to stigma and hate speech since the beginning of the COVID-19 pandemic. This is particularly evident on social media [1]. The experience with previous epidemic diseases, such as Ebola and HIV, shows that this behavior against victim populations not only is unfair and inhumane but also leads to secondary harms, such as the isolation of communities; increased depression, anxiety, and rates of suicide; and other social issues, such as discrimination in employment rates, bias in education systems, and various other adverse chain effects [2]. The necessity of timely and large-scale intervention in this situation is, therefore, obvious and unequivocal. We present a data set of stigmatizing tweets and also demonstrate how well the state-of-the-art techniques for text categorizing perform on our data set.

Constructing a data set containing stigmatizing language against people of Asian descent influenced by the COVID-19 pandemic is important from several aspects. First, it confirms the existence of such incidents and emphasizes the necessity of awareness-raising initiatives by professionals. Second, it is a general call to invite researchers from different domains, including psychologists, public policy authorities, and sociologists, to dive deep into the problem; analyze the complex economic, political, historical, and cultural underlying roots of anti-Asian stigmatization and hateful behaviors; and ultimately provide multifaceted solutions for eliminating unfair behavior against the Asian populations during and especially in the long transient time after the pandemic.

Third, a labeled data set provides an opportunity for a qualitative analysis of the existing discourse about COVID-19 and its relevance to communities of Asian descent. Finally, it equips computer scientists with the first element needed for building an algorithm to automatically detect COVID-19–related stigmatizing and hateful language (ie, a high-quality cleaned and labeled data set).

### Stigma

In the past years, there has been a growing body of work studying online stigma directed toward the Asian population worldwide. Although many studies focus on vaguely defined hate on social media during COVID-19, they lack a more theoretically grounded approach. This requires rethinking the term “hate speech.” Although the term “hate speech” is used colloquially in online spaces, we find the term “stigma” to be more well defined in the literature, and it is often seen to be the root cause of online hatred [3,4]. Although we do not claim stigma to be the reason for all hatred, based on established social science research on stigma, we see it as 1 of the drivers of hatred toward a particular population as a result of a problematic sociopolitical context. Having said that, the definition of stigma itself has undergone changes from stigma being “a person’s attribute” to a broader “social condition.” For stigmatization to occur, power must be exercised [5]. Various scholars have shown that the more powerful part of a group often stigmatizes the powerless counterpart who might pose a threat to them. Michelle Foucault’s [6] celebrated work on madness depicts this vividly by showing how people with mental illness were stigmatized and imprisoned to ensure their obedience to the state-imposed law and order in France. At various points in history, stigma toward mental health problems, as well as toward contagious diseases, was used as a political tool shaped for the benefit of a dominant group at the expense of “others” [7-9]. In this study, we form our understanding of hatred toward Chinese people on social media during COVID-19 through this lens and understand what constitutes this stigma.

### Literature Review

Several scholars have shown how the COVID-19 pandemic saw a significant surge in anti-Asian stigma (Sinophobia) on social media platforms, such as Twitter and Reddit [10]. There have been a number of works that present various machine learning (ML) techniques to identify Sinophobic content [11].

Some researchers have published large data sets of tweets related to COVID-19 [12-14]. Other works have provided manually annotated Twitter data and created comprehensive questionnaires that can be used for the classification of future tweets by volunteer groups [15,16]. Ziems et al [17] presented the COVID-19-HATE data set and showed the occurrence of counterhate tweets in the early stage of the pandemic. When detecting hate speech on social media, the first step for many is to differentiate hateful posts from nonhateful posts. Waseem et al [18] suggested that this process can be approached by crowdsourcing annotators. De Gibert et al [19] created a hate speech data set from a White supremacy forum composed of thousands of sentences manually labeled as containing or not containing hate speech.

The detection techniques proposed in the literature often use state-of-the-art models from the field of ML and natural language processing (NLP). These models are trained on large manually annotated data sets. Complex neural networks, such as the Bidirectional Encoder Representations from Transformers (BERT) model, have been shown to work well on publicly available data sets annotated for racism, sexism, hate, or offensive content on Twitter [20]. Huang et al [21] addressed the issue that many models can pick up on human biases and become discriminatory. In combining previously published corpora, they created a multilingual hate speech corpus that also provides author attributes, allowing for future research to be conducted with more fairness.

### Our Work

We present 1 such data set from our work with 11,263 tweets in the English language that are manually annotated into predefined categories. This data set can be used as a starting point for further qualitative research, as well as a benchmark for designing stigma detection algorithms. The data set was generated after 3 iterative stages of manual hashtag extraction, topic modeling and keyword extraction, keyword-based scoring, and, finally, human labeling. In addition to releasing the data set, we developed baseline ML models for the automatic detection of sensitive COVID-19–related hate speech and stigma in text data. We trained support vector machine (SVM)+stochastic gradient descent (SGD), Gaussian naive Bayes, random forest, AdaBoost, and multilayer perceptron classifiers on the vectors of term frequency–inverse document frequency (TFIDF) language-based features with the best accuracy of 73% by the SVM+SGD. To evaluate the performance of the state-of-the-art deep learning classifiers on this task, we also fine-tuned the BERT language model on this data set, which resulted in an accuracy of 79%. The data set and instructions for labeling can be viewed in the Github repository [22].

In the next sections, we will explain the details of data collection, data annotation, and content analytics. We will also present and compare the results of the experiment with the ML models, which are designed to detect stigmatizing language against people of Asian descent.

## Methods

As already discussed, creating such a data set is challenging for various reasons. We devised an iterative method for selecting a final set of tweets that we manually annotated.

### Ethical Considerations

This study was conducted under the ethics protocol approved by the Research Ethics Board (REB) of the University of Toronto, Canada (Protocol# 42786). There were a few special measures taken to ensure the ethical standards of the study. First, our study did not involve collecting data directly from social media. Instead, we used data that were collected and shared for public use and research by other research organizations [12-14]. Second, in our research, we did not use any personally identifiable information. None of our analyses and corresponding results report any individual or their expression. All the findings reported in this study are cumulative. The produced data set is also cumulative data, which is a subset of already published data sets. Third, our study made sure none of the researchers involved in this project was physically or emotionally hurt by the study. All the encoders were paid according to the fair pay standard of the city. Finally, we discussed the findings with the representatives of Chinese communities through the Chinese-Canadian National Council for Social Justice (CCNC-SJ) to ensure that we voice their communal concerns.

### Challenges of Creating a Stigma Data Set

Constructing a data set of stigmatizing tweets is a challenging task for a number of reasons.

First, the number of stigmatizing tweets makes up a small fraction of any Twitter data set. In addition, manual labeling itself is an expensive process. It would be infeasible to label thousands of tweets if there is not a high probability that the selected subset of data contains a significant number of potentially stigmatizing tweets. When we consider the size of the original data set (668 million), finding the stigmatizing tweets would be like finding a needle in a haystack.

Second, even with a selected set of tweets that have a high probability of producing stigmatizing tweets, stigma itself is highly subjective, and different people may perceive different things to be offensive to their culture. It is also important to have good-quality annotators; particularly desirable would be to have the annotators identify as belonging to the target ethnicity. We tried to leverage crowdsourcing platforms, such as Mechanic Turk, but the process proved frustrating with low-quality labels that were not trustworthy. Hence, our choice of annotators was important and cheap solutions for labeling did not work in our case.

Moreover, among diverse Chinese populations, it is unlikely that there would be consensus on whether particular content is perceived to be stigmatizing. It is desirable to have an idea of how much opinions vary, and possibly have multiple labels to poll for a final label that is of a higher quality than if only a single annotator were to do it.

For these reasons, we used an iterative approach to constructing our data set so we could filter tweets to a potentially stigmatizing set before finally choosing to manually label them. Moreover, we performed most of the labeling through Chinese volunteers from the Chinese Social Justice Association at the University of Toronto.

### Iterative Data Set Construction and Data Analysis

We generated a high-quality data set of stigmatizing tweets through a series of iterations. Our goal was to sift through 668 million tweets related to COVID-19 that we compiled and arrive at a more manageable number that we could then manually annotate.

**Figure 1:**
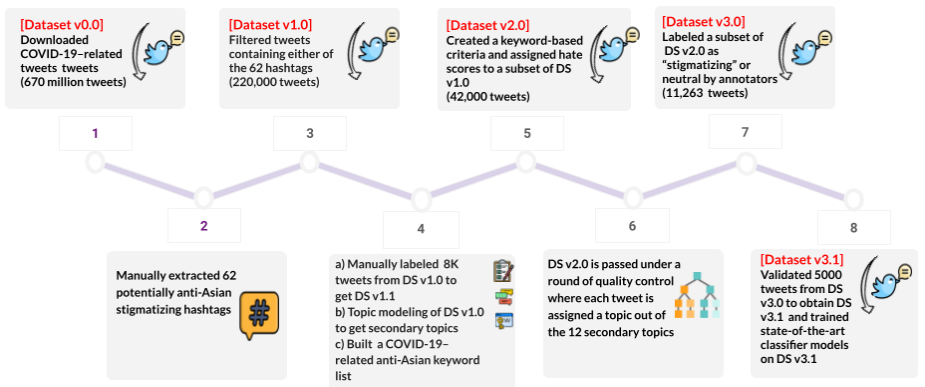
Flowchart detailing the key steps of our work.

Figure 1 shows a high-level summary of how we generated our final data set. We downloaded 670 million COVID-19–related tweets and finally arrived at a high-quality *Data Set v3.0* containing 11,263 tweets labeled as stigmatizing, nonstigmatizing, or unknown/irrelevant. The key steps involved included manual hashtag extraction, topic modeling, and multiple rounds of manual labeling. We further validated 4998 (44.37%) tweets from Data Set v3.0 to get *Data Set v3.1* and ran state-of-the-art classification models on Data Set v3.1 for automatic detection of stigmatizing text. The steps involved are discussed later in detail.

#### Downloading an Assembly of 3 Existing Data Sets of COVID-19 Tweets

We downloaded COVID-19 Twitter data sets collected by Chen et al [12], Banda et al [13], and Gruzd and Mai [14] and combined 668 million English tweets without duplicates from these 3 sources that were publicly shared on Twitter from January 21 to July 31, 2020, and contained general terms relevant to the COVID-19 pandemic, including but not limited to “COVID-19,” “coronavirus,” “CDC,” “NCov,” “SARS-CoV-2,” and “pandemic.” For further details regarding data collection, please refer to the papers cited before [12-14]. The Twitter data sets contained tweet IDs that we had to hydrate (process of getting full tweets from tweet IDs) to get a full body of tweets. We used multiple accounts to speed up tweet hydration (since Twitter rate-limits their application programming interface [API]) and stored 668 million tweets in a MySQL database. We called this *Data Set v0.0*.

#### Preparing a Data Set Based on 61 Potentially Anti-Asian Stigmatizing Hashtags

To generate a subset of Twitter data containing anti-Asian content, we decided to use a list of hashtags that might give us potentially stigmatizing tweets. To come up with such a list of hashtags, we worked with the Chinese Social Justice Association at the University of Toronto to identify 61 hashtags, which led to potentially anti-Asian stigmatizing and hateful content. A list of co-occurring hashtags was found computationally, and then, the most relevant ones were chosen manually from that list. Eight members of that association, each representing Chinese origin and culture, identified the hashtags, such as #CCPVirus, #antichinazi, and #madeInChinaInfected. The complete list of hashtags can be seen in Multimedia Appendix 1.

After filtering for these specific hashtags and narrowing further to include only English language tweets that also contain location data, we obtained tweets more specific to the online discourse about the pandemic and its relationship to people of Asian heritage, especially the Chinese. We called this collection of 220,000 tweets *Data Set v1.0*.

Although we used location data as a filter criterion for this step, we did not do so for our final data set. So, any bias this step might have introduced was negligible, since this step was used as an exploratory step to understand our data better. We obtained our final data set without filtering by location.

**Figure 2:**
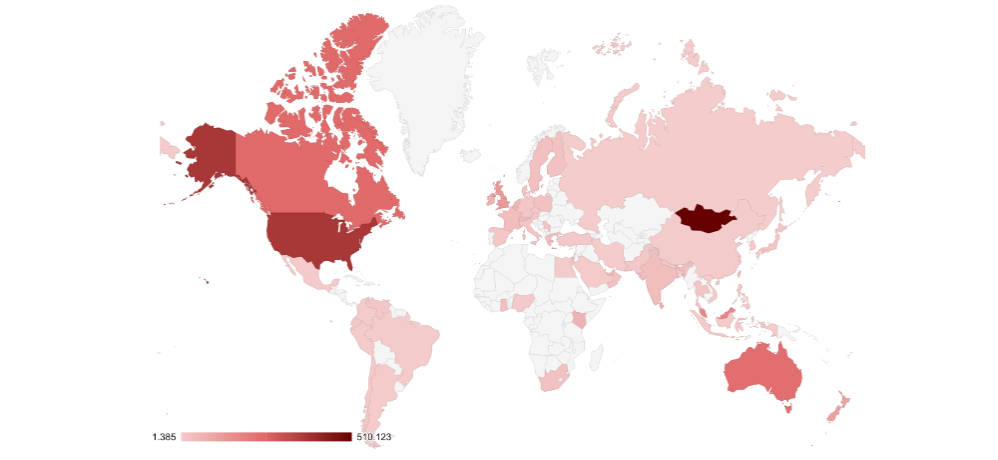
Location-wise distribution of tweets in Data set v1.0.

Figure 2 shows the location-wise distribution of tweets in Data Set v1.0. A significant number of tweets are from Mongolia. This could be because of an anti-Chinese political wave there or because the troll authors used a Mongolia virtual private network (VPN) to access the internet.

#### Manual Labeling, Keyword Selection, and Topic Modeling

We wanted to dive deeper into Data Set v1.0 to better understand the nature of the discourse around people of Asian heritage and COVID-19. We, therefore, sampled a random subset of 8745 tweets from Data Set v1.0, asked 2 annotators to inspect the tweets, and assign them 1 or more of the following categories: stigmatizing, antistigmatizing, political, news/facts/misinformation, and other topics. The definition of each category is given next. The categories were decided through discussion between the annotators and our researchers.

- Stigmatizing: These are tweets that attribute a negative characteristic to the general community of Asians, especially people of Chinese nationality.
- Antistigmatizing: These are tweets that explicitly oppose the stigmatizing attitude toward the Asian community.
- News/information/misinformation: These tweets do not fall under either of the first 2 categories and state some kind of data related to COVID-19; the data can be valid or invalid. Examples could be the number of patient cases in different countries, travel and airport situations, information about vaccine production, health care instructions, etc.
- Political: This category encompasses tweets talking about political affairs related to the COVID-19 pandemic. The majority of the discussions in this category are about the Chinese government (Chinese Communist Party [CCP]), its role in the pandemic, and the way it responded to the crisis. However, the CCP is not the only topic of discussion in this category; other subjects are Donald Trump, World Health Organization (WHO), the Centers for Disease Control and Prevention (CDC), the Hong Kong government, the Muslims of China, etc. It is worth mentioning that the tweets against the Chinese government only are not necessarily considered stigmatizing unless mixed with a stigmatizing or racist sentiment against the general people of Chinese nationality or Asian heritage.
- Other: Finally, the fifth category contains tweets that do not fit in any of the previous categories. The content of this category is mostly neutral opinions, prayers, questions, advertisements, expressions of fear or hope, or other general COVID-19–related conversations without a position for or against the Asian population.

Once the 2 annotators categorized the 8745 tweets, a validator resolved the discrepancies between the first 2 annotators. We called this data set *Data Set v1.1*. We show some examples from the data set in Table 1.

We next compiled a list of high-frequency words from our set of stigmatizing tweets from Data Set v1.1. The list of these keywords can be seen in Textbox 1.

Data Set v1.0 and Data Set v1.1 are valuable; however, a significant percentage of the data is still unrelated to the topic of our interest. Furthermore, since the inclusion criterion was chosen based on 61 specific hashtags, a considerable portion of the tweets ending up in Data Set v1.0 and Data Set v1.1 was identified to have been originated by troll authors or spam posts as per annotator labels. Around 1506 (28%) of all 8745 labeled tweets were spam, ads, or troll posts that just contained the hashtags possibly to piggyback off on the virality of the selected hashtags. Therefore, this iteration of our data was not of sufficiently high quality, and we used insights from this step to refine our selection of tweets for annotation.

In addition to manual labeling of the tweets, we applied the Twitter–latent Dirichlet analysis (Twitter-LDA) topic modeling algorithm [23] to Data Set v1.0 to automatically identify the most prominent subtopics discussed online related to COVID-19 and the Asian population. Twitter-LDA is a variant of LDA [24], which is customized for the text data in the size of a tweet. We identified the most prominent topics mentioned in the COVID-19–related anti-Asian discourse, as shown in Textbox 2.

**Table 1:** Examples of labeled tweets from Data Set v1.1.

Category	Example tweet
Stigmatizing	I think China is onto a theme here - Bat....Cat....Rat in fact every animal gets eaten in #chinazi. No wonder the most lethal viruses come from there! #coronavirus #coronaviruswuhan https://t.co/JwxbT10h8D
Antistigmatizing	Director of WHO praising China’s response to the virus and saying the actual risks are posed by other countries, but our racist news media will continue hystericizing China &amp; enabling more anti-Asian racism from Westerners in the process
News/information/misinformation	#Breaking From 10 am on January 23, the city's urban bus, subway, ferry, and long-distance will be suspended; for no special reason, citizens should not leave Wuhan. The airport and train station departing from Wuhan will be temporarily closed. #WuhanOutbreak #WuhanCoronavirus https://t.co/agjLVccI9E
Political	@washingtonpost I am afraid that the real situation is much worse as evidenced, It has already been widespread. #CCP can't be trusted as their news have been censored. They control the information regardless of their ppl's deaths. #WuhanCoronavirus #antichinazi https://t.co/QoKxykdkFM
Other	Talking about two immune-boosting foods to help build body's resistance against the #Wuhancoronavirus Stay safe, stay strong! Watch the full video here: https://t.co/oqtKwYJBAF https://t.co/6117sydKGp

**High-frequency words in anti-Asian-stigmatizing tweets.**

bat, animal, eating, eat, snake, dog, rat, soup, cat, wild, critter, swine, pig, meat, wet, market, kill, killer, killed, killing, made in china, plague, fatality, threat, guilty, pay, boycott, lie, lied, liar, cover, covering, silenced, trust, trusted, coverup, conceal, concealed, deception, transparency, hoax, biological, weapon, bio, war, warfare, biowarfare, lab, leaked, deliberately, stealing, dictator, tyranny, communist, posed, threat, fear, guilty, guard, army, military, murderer, boycott, imprisoned, sanction, genocide, crippling, died, censorship, freedom, fight, fighting, f*k, f**king, sh*t, b****rd, evil, sewage, racism, racist, suffering, blame, blamed, threaten

**The most prominent topics related to the COVID-19 pandemic and people of Asian descent.**

1. Wet market and eating habits
2. COVID-19 cases
3. Bioweapon
4. Wuhan virus lab
5. The Chinese government (Chinese Communist Party [CCP])
6. Antistigmatizing (talking against hate speech and racism)
7. News
8. Information or misinformation
9. International politics
10. Donald Trump
11. Chinese Uyghur Muslims
12. Other

#### Creating a Score-Based Search Criterion to Construct a Higher-Quality Data Set

Using the insight gained from the keywords and topic modeling result from Data Set v1.1, we came up with a new criterion for preparing a new version of the data set without the aforementioned shortcomings. We also took care to get our new data set version labeled by members of the stakeholder community (ie, Chinese). First, we derived a score-based criterion for evaluating the potential degree of anti-Asian stigma and hate speech in each tweet in Data Set v0.0. We did this using a keyword-based approach, where we used the high-frequency word list presented in Textbox 1 as keywords. We assigned a score to each tweet by counting the number of keywords that occur in it. In addition, only tweets that contain 1 of the words “china,” “chinese,” “Asian,” and “cpp” were included in the query, since we were only interested in the content concerning the Chinese and more broadly the Asian population.

For example, if a tweet contains 5 keywords, it gets a score of 5, and if there is only 1 keyword in a given tweet, it gets a score of 1. It is important to mention that duplicate keywords were counted only once (ie, the score was based on distinct keywords).

Before calculating the score for each tweet, both the Tweet body and keywords were preprocessed. The preprocessing steps involved tokenizing the text, removing the noise characters, and lemmatizing the words. In addition, some keywords were combined to appear as pairs of words, such as “wet market,” “eat animal,” and “bio weapon.”

Finally, we selected all COVID-19–related tweets with a score >3 (ie, ≥4). In other words, we assigned a presumed score to each of the tweets in Data Set v0.0, which included 1 of the words “china,” “chinese,” “Asian,” and “cpp,” and then selected the tweets with a score >3. This data set contained 21,752 tweets with a potential stigma score >3.

In addition to this set of tweets, we also randomly sampled the same number of COVID-19–related tweets with a score of 0 (N=21,752). This part of the data most probably has nonstigmatizing content and was included in the data set to act as control data. This data set was called *Data Set v2.0*.

#### Labeling the Score-Based Data Set (Data Set Quality Control)

We did not assign the tweets having a score >3 in Data Set v2.0 a “stigmatizing” label yet. We wanted to pass the tweets through a round of quality control to make sure the underlying concept in the tweets was stigmatizing.

**Figure 3:**
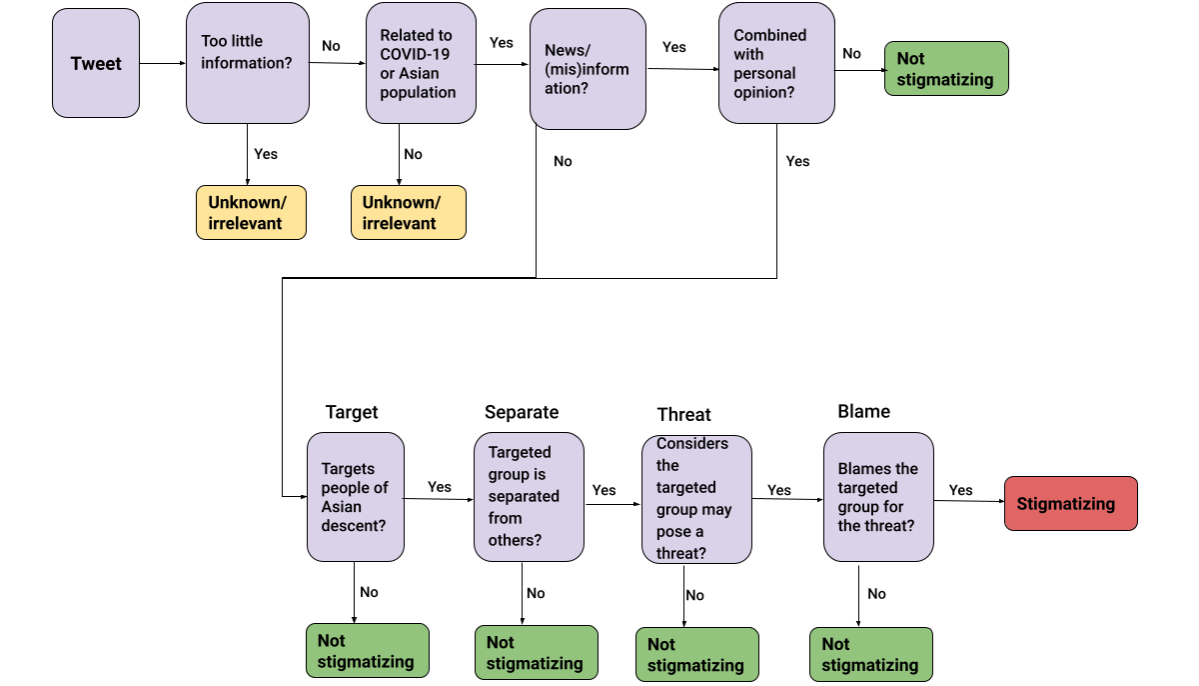
Summary of guidelines followed by annotators while labeling tweets.

Therefore, we reached out to 16 volunteers from the Asian community and asked them to investigate the content of a subset (~12,000 tweets) of Data Set v2.0 and assign each tweet a “stigmatizing,” “nonstigmatizing,” or “unknown/irrelevant” label. Tweets containing too little information or tweets unrelated to COVID-19 or the Asian population were labeled as unknown/irrelevant. Tweets targeting and blaming the Asian population specifically were labeled as stigmatizing. The volunteers were further asked to categorize the stigmatizing tweets into low, medium, or high groups, depending on the extent of stigmatization. The rest of the tweets containing news/misinformation or personal opinions were labeled as nonstigmatizing. The volunteers followed detailed instructions to assess the content of each tweet; they were also given some examples to master the task. Figure 3 shows a summary of the guidelines followed by the annotators while labeling the tweets. The detailed instructions can be viewed in our Github repository.

They were also asked to classify the subcategory of each tweet by assigning it to 1 (8%) of the 12 categories in Textbox 2. Finally, the volunteers could report any additional recurring topic that they identified in the data set. As an outcome of this step, we had a data set of 11,263 tweets that were labeled as stigmatizing (positive), nonstigmatizing (negative), or irrelevant (unknown). This data set, which was labeled by human annotators, was named Data Set v3.0.

#### Validating Labels (Further Quality Control)

Data Set v3.0 can be considered to be a high-quality data set of stigma tweets during COVID-19. Next, we wanted to perform another round of labeling to get an idea of how much variance there can be across multiple labelers. We randomly sampled 4998 tweets from Data Set v3.0 and had another annotator (again from the Chinese student body) label them. For the sake of convenience, we only considered stigmatizing versus nonstigmatizing to find our agreement scores between annotators. The interannotator percentage agreement between the first and second rounds of annotators came to 66%, with a Cohen κ coefficient [25] of 0.33 (fair agreement). It is worth noting that the agreement scores were not too high. There are a number of reasons we think these discrepancies existed between the 2 annotators. A big reason is that stigma is highly subjective. The same tweet can be considered stigmatizing to some people and not stigmatizing to others despite being given the same criteria for classification. At this stage, all labeling was performed by annotators who belonged to the same attacked group (Asian descent), so we think it was useful to have these labels despite the disagreements.

The discrepancies between the first set of annotators and the second annotator were resolved by a third annotator (another Chinese student), who freshly labeled the tweets where disagreements occurred. The final annotator’s decision on the discrepancies were used as the final label for those 4998 tweets. We called this Data Set v3.1, and this was our highest-quality stigma data set. Data Set v3.1 and Data Set v3.0 had a percentage agreement of 75% and a Cohen κ coefficient of 0.52 (moderate agreement).

When we asked the validator why they thought there was a somewhat high degree of disagreement, they mentioned differences in opinion on tweets that:

- Are anti-CCP; such tweets are not considered to be stigmatizing unless they also blame the Chinese people according to our instructions
- Mentioned animals and pointed out how different people considered different things to be offensive based on age, geography, background, socioeconomic conditions, etc

We considered Data Set v3.0 to be a moderately high-quality data set and Data Set v3.1 to be a more refined, high-quality data set.

### Analysis of Data Set v3.0

**Figure 4:**
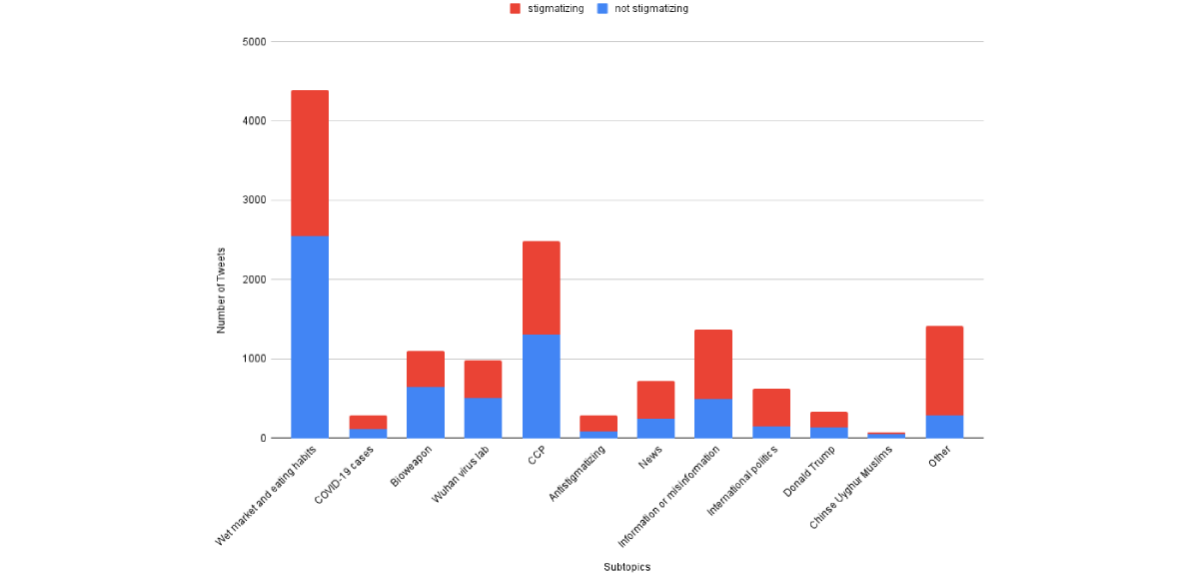
Distribution of topic-wise stigmatizing and nonstigmatizing tweets. CCP: Chinese Communist Party.

Here, we provide some data set statistics to help understand our Data Set v3.0 better. The human annotators were asked to assign each of the stigmatizing and nonstigmatizing tweets to 1 (8%) of the 12 topics, as given in Textbox 2. Figure 4 shows the distribution of topic-wise stigmatizing and nonstigmatizing tweets. The most popular COVID-19–related topic is “Wet Market and Eating Habits,” containing the highest number of both stigmatizing and nonstigmatizing tweets.

**Figure 5:**
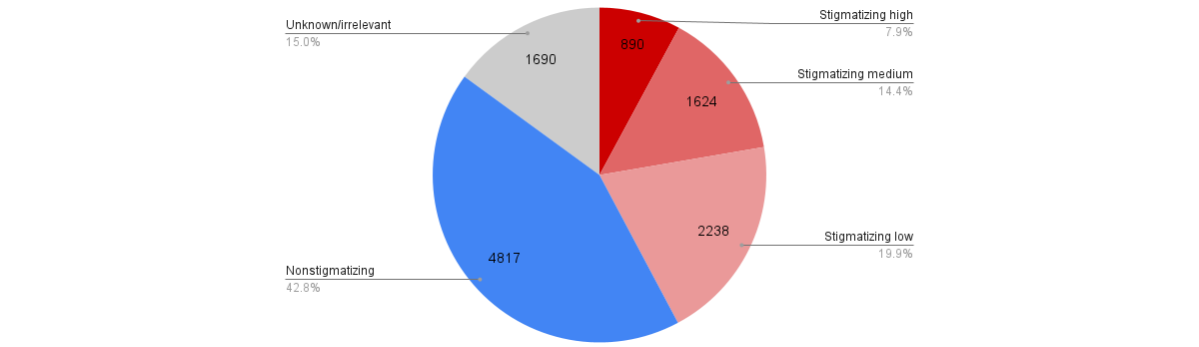
Distribution of stigmatizing (and their extent) and nonstigmatizing tweets.

Looking at the percentage of different categories of tweets from Data Set v3.0 (Figure 5), we found that 4754 (42.21%) tweets are stigmatizing, 4818 (42.78%) tweets are nonstigmatizing, and 1691 (15.01%) are unknown/irrelevant tweets. Of the 4754 stigmatizing tweets, 2239 (47.10%) were categorized as low, 1625 (34.18%) as medium, and 890 (18.72%) as high with respect to their extent of stigmatization.

#### Sentiment Analysis

We measured the polarity and subjectivity of the stigmatizing and nonstigmatizing tweets using Python’s textblob [26] library. Table 2 shows the category-wise average polarity and average subjectivity.

We found that the stigmatizing tweets have an overall negative sentiment, particularly in comparison to the nonstigmatizing tweets.

**Table 2:** Category-wise average polarity and average subjectivity.

Label	Polarity	Subjectivity
Stigmatizing	–0.084091	0.549432
Nonstigmatizing	0.700000	0.400000

#### Frequent Hashtags

We extracted hashtags from the tweets and created word clouds (Multimedia Appendix 2) to understand the stigmatizing tweet topics better and to view the frequently used hashtags these tweets used. We excluded the keywords used to filter the tweets (eg, “chinesevirus,” “wetmarket,” “bioweapon,” “ccp”).

We found that the stigmatizing tweets have hashtags targeting the Chinese (and broadly Asian) community from Multimedia Appendix 2. Some of the highest-frequency stigmatizing hashtags include *#chinaliedpeopledied*, *#boycottchina*, *#ccpchina*, *#chinacoronavirus*, *#makechinapay*, and several variations of these.

#### Pointwise Mutual Information

We calculated the pointwise mutual information (PMI) [27] value for each word and pair of words (bigram) toward the stigmatizing and nonstigmatizing categories, as shown in Tables 3 and 4, respectively.

From Tables 3 and 4, we can see that the top unigrams and bigrams associated with the stigmatizing and nonstigmatizing tweets differ significantly. The stigmatizing tweet topics mostly revolve around the Chinese community's eating habits or make derogatory comments against the Chinese population.

**Table 3:** Top 10 words in stigmatizing and nonstigmatizing tweets based on PMI^a^ scores.

Stigmatizing		Nonstigmatizing	
Words	PMI	Words	PMI
dirty	0.270769	bloomberg	0.165349
chinesevirus	0.270188	iran	0.157493
barbaric	0.268468	russia	0.155230
insects	0.241584	hoax	0.152316
disgusting	0.241122	blames	0.143863
filthy	0.231158	news	0.143725
bastards	0.228796	testing	0.140990
nasty	0.227321	test	0.137785
alive	0.226920	support	0.134680
mice	0.224538	vaccine	0.131006

^a^PMI: pointwise mutual information.

**Table 4:** Top 10 pairs of words (bigrams) in stigmatizing and nonstigmatizing tweets based on PMI^a^ scores.

Stigmatizing		Nonstigmatizing	
Bigrams	PMI	Bigrams	PMI
innocent animals	0.318808	silencing bloomberg	0.2379
dogs alive	0.301495	soup narrative	0.2379
cat bat	0.301138	family financially	0.2379
eat shit	0.297512	dependent vast	0.2379
eating shit	0.295743	devastate family	0.2379
fuck china	0.290280	financially didnt	0.2379
rat bat	0.289913	nda silencing	0.2379
eating chinese	0.275221	didnt sign	0.2379
eat like	0.275221	story critical	0.2379
dog bat	0.274254	sign nda	0.2379

^a^PMI: pointwise mutual information.

## Results

### Primary Contributions

Our primary contributions presented in this paper are the labeled data sets. Data Set v3.0 has 11,263 tweets with primary labels (unknown/irrelevant, not-stigmatizing, stigmatizing-low, stigmatizing-medium, stigmatizing-high) and tweet subtopics (eg, wet market and eating habits, COVID-19 cases, bioweapon). Data Set v3.1 contains 4998 (44.37%) tweets randomly sampled from Data Set v3.0, with multiple labels, as discussed in the previous section.

One of the main goals of preparing a high-quality reliable data set of anti-Asian COVID-19–related content is to provide computer scientists with the material needed to train a classifier for automatic detection of anti-Asian stigma in text-based settings. In this section, we use our Data Set v3.1, containing 3343 (66.89%) positive (stigmatizing)–labeled and 1655 (33.11%) negative (nonstigmatizing+unknown/irrelevant) –labeled tweets, to build several baselines and state-of-the-art classifier models for automatic detection of hate speech.

### Preprocessing Tweets

The tweets, as given, were not in a form amenable to feature extraction for classification as there was too much noise. We preprocessed the tweet texts as follows:

- Replacement of all nonspace whitespace characters, including newlines, tabs, and carriage returns, with spaces
- Replacement of HTML character codes with their ASCII equivalent
- Removal of URLs
- Removal of duplicate spaces between tokens
- Lemmatization and sentence segmentation using spaCy [28]

After these steps, we ran feature engineering on the clean tweet texts.

### Feature Engineering

The TFIDF vector was calculated for each preprocessed tweet to serve as the input feature vector for the classic supervised learning algorithms. TFIDF is a numerical statistic that is intended to reflect how important a word is to a document in a collection or corpus, based on its normalized repetition frequency.

On a different thread, the tweet texts were tokenized, padded, and converted to unique IDs. The final ID tensors were used later as inputs to a BERT-based classifier. BERT [29] is a transformer-based deep learning technique for NLP developed by Google.

#### Models

The TFIDF feature vectors were used to train the following classifiers:

- SVMs [30] with SGD [31]
- Gaussian naive Bayes [32]
- Random forest [33]
- AdaBoost [34]
- Multilayer perceptron (neural networks) [35]

Next, the unique word ID tensors from the previous steps were used in fine-tuning a pretrained BERT-based classifier.

The classifiers were trained to classify the tweets as stigmatizing or nonstigmatizing. The performance metrics of the models are provided in Table 5. We reported the weighted average metrics after several runs. The BERT-based model gave an accuracy of 79%, with an F-score, calculated as 2 × [(precision × recall)/(precision + recall)], of 0.70.

**Table 5:** Classification model results on Data Set v3.1.

Classifier	Accuracy	*F*-score	Recall	Precision
SVM^a^+SGD^b^	0.73	0.71	0.73	0.72
Gaussian naive Bayes	0.72	0.70	0.72	0.71
Random forest	0.70	0.64	0.70	0.73
AdaBoost	0.70	0.70	0.70	0.69
Multilayer perceptron	0.70	0.70	0.70	0.69
BERT^c^	0.79	0.70	0.67	0.73

^a^SVM: support vector machine.

^b^SGD: stochastic gradient descent.

^c^BERT: Bidirectional Encoder Representations from Transformers.

## Discussion

### Principal Findings

We presented a data set containing 11,263 tweets that contained 4754 tweets potentially stigmatizing toward the Asian community and 6509 neutral (nonstigmatizing+ unknown/irrelevant) tweets. This was our Data Set v3.0. A subset (4998 tweets) of Data Set v3.0 was validated, and we called this Data Set v3.1. We trained our classification algorithms against Data Set v3.1 and obtained the best accuracy of 79% using the BERT-based model.

This data set is our primary contribution, as well as the methodology used in preparing the data set. It is a challenging task to construct a high-quality data set of hate speech directed toward a marginalized group from Twitter data. We believe our approach can serve as a good example of how this is possible.

### Comparison With Prior Work

Several works have shown the relationship between online public sentiment and public health [36-39]. Lloret-Pineda et al [40] identified different types of racism (eg, individual [both active and inactive] and cultural racism) displayed on Twitter during the first quarter of 2020 toward Asians. Moreover, they recognized the kinds of responses from both advocacy and activism from the common Twitter users against these racist sentiments. Budhwani et al [10] showed how COVID-19 stigma is likely being perpetrated on Twitter by referencing the virus as “Chinese virus” or “China virus.” Logie et al [41] suggested possible ways to solve tensions between COVID-19 containment and stigma mitigation. Our data set was developed considering the findings from prior works.

### Limitations

Overall, our choice of the final data set is likely biased toward a certain distribution and may not be representative of all kinds of stigma directed toward the Chinese population during COVID-19. This is unavoidable, given the strategy we picked of using hashtags or keywords to narrow down our search, as well as picking only English language tweets. We also applied a time constraint to our analysis, as we looked at the first phase of the pandemic. It is possible that some data characteristics might have changed as the pandemic progressed, such as new stigmatizing hashtags, keywords, and emergence of new topics.

Another issue we faced was a discrepancy in labeling between different annotators and inconsistency between labels for the same annotator as time progressed, which ultimately resulted in a lower-than-average interannotator agreement score. This further shows how subjective the idea of hate/stigma is. Here is an example:

I've said from day #1 that COVID-19 is a Bio-weapon manufactured by China and supported and paid for by Bill Gates, George Soros and supported by Democrats. Not from eating bats. Time to lay some smack on China and those involved and make them pay dearly.

We found various similar examples that reference COVID-19 as a bioweapon or other conspiracy theories where annotators differed in their views on whether they are stigmatizing. For our purposes, we believe our approach is sufficiently rigorous and we ought to trust the subjectivity of our annotators and their unique experiences as targets of said stigma. As such, we leave room for a higher disagreement score.

Moreover, it is difficult to identify the underlying intention behind sarcastic tweets, even by human annotators. Here is an example of a sarcastic tweet that our annotators from Data Set v1.1 incorrectly labeled as nonstigmatizing. The tweet can be understood to be sarcastic by the last line, where the user uses a derogatory term to address Chinese people, which completely undermines the validity of the tweet as a defense of the Chinese people.

It’s absolutely racist to call the CHINESE VIRUS as #ChineseVirus Even though it was originated from China, you cannot blame China for this #ChineseVirus19 and Calling all the as***les of China as #chinaIsAs***le is racist and xenophobic.

### Future Research

In the future, we hope to address the problem of discrepancies in labeling that were demonstrated in the labeling process in this study. As we have seen, there were often debates and disagreements on whether a speech was hateful. A possible solution could be embracing the “jury learning” approach that was recently proposed by Gordon et al [42], which aims to produce a multiscale hate value for a speech instead of classifying that as hateful. This approach could be a good solution to the high interannotator disagreement score problem. Additionally, we want to further this study by training the classification algorithms such that they can capture various subtleties in informal social media texts, such as sarcasm (an example of which is found in the “Limitations” section). Another future direction can be building and maintaining a dynamic data set to capture the temporal variations of stigmatization on social media and keeping track of the evolution of stigma toward a particular group over time. For example, it might be interesting to see which phrases are used over time to stigmatize Asian people and what events might have motivated them. This can shed further light on the nature of online hate and stigma, how it propagates, what language it assumes, etc. Such analysis can give us valuable insights that can help design interventions and improved detection techniques.

### Conclusion

Our main contribution of this paper is a high-quality data set of COVID-19 stigma toward Asian people that was manually annotated and carefully compiled. We reaffirm the existence of said stigma and further emphasize the need for timely intervention. Our data set will be publicly accessible and can benefit researchers from various domains to study how stigma propagates in online spaces and the language it assumes. Twitter particularly features short-form text and informal language, which has characteristics different from long-form text or media that use more structured language. It is important to study the language of social media for tackling similar research problems, and our data set can be a useful tool to this end. Taken together, through multidisciplinary research, we can arrive at multifaceted solutions for eliminating hateful and unfair behavior against the Asian population during and especially in the long transient time after the pandemic.

We believe our work can help predict and hence reduce the stigma, hate, and discrimination against Asian people during future crises like COVID-19. Moreover, stigma shares a lot of common characteristics across various targets. It can be useful to study the relationship between stigma and crisis, which can help us infer such developments early. Early detection of such discourse can help mitigate future catastrophic events. The misinformation and stigma surrounding a pandemic like COVID-19 can severely harm public health efforts owing to mistrust by the marginalized communities or can lead to hate crimes. Hence, it becomes important to identify and put a stop to the hate speech and stigmatization at an early stage.

A labeled data set also provides an opportunity for more qualitative analysis of the conversations surrounding COVID-19 and anti-Asian stigma. In addition, it allows for quantitative analysis and application of state-of-the-art models for detecting anti-Asian stigma on social media.

## Appendix

Multimedia Appendix 1 List of 62 hashtags used to create Data set v1.0.

Multimedia Appendix 2 Word cloud of hashtags from stigmatizing tweets.

## Abbreviations

BERT: Bidirectional Encoder Representations from Transformers
CCP: Chinese Communist Party
LDA: latent Dirichlet analysis
ML: machine learning
NLP: natural language processing
PMI: pointwise mutual information
SGD: stochastic gradient descent
SVM: support vector machine
TFIDF: term frequency–inverse document frequency

## Data Availability

We made our data publicly available and accessible [22]. If the link is in any way compromised, we urge researchers to reach out to us.
