# Supplementary material for: Development of a COVID-19–Related Anti-Asian Tweet Data Set: Quantitative Study
Source: JMIR Form Res. 2023 Feb 28;7:e40403. doi: 10.2196/40403 (PMC9976773; doi:10.2196/40403)
Supplement: Multimedia Appendix 1 [file formative_v7i1e40403_app1.docx]

**Multimedia Appendix 1**

**Table S1.** List of 62 hashtags used to create Data set v1.0.

| #CCPVirus | #coronaindia | #Chinacoronavirus | #MacauVirus |
| --- | --- | --- | --- |
| #wuhanvirus | #coronavirussingapore | #coronajihad | #carbonaravirus |
| #BoycottChina | #antichinazi | #anticcp | #ChinaCoronaViru |
| #coronavirususa | #ChinesePneumonia | #wuhanvirus2020 | #wuhanviruscoverup |
| #WuhanOutbreak | #VirusChina | #BeijingVirus | #therealwuhanvirus |
| #coronaviruswuhan | #WuhanSARS | #boycottmadeinchina | #chopstickflu |
| #WuFlu | #ChinaLied | #aliexpress | #China_Make_Corona_in_Lab |
| #CCP_is_terrorist | #FuckChina | #ChinaCorona | #VirusWuhan |
| #coronavirusjapan | #ChinaziVirus | #communistvirus | #WuhanSARS¬† |
| #Chinesevirus | #Kungflu | #wuhansars | #ChinaPneumonia¬† |
| #China_is_terrorist | #chinaliedpeopledied | #Chinapneumonia | #wuhanviruscorona |
| #wuhanflu | #madeinchina | #wuhanvirusoutbreak | #CommunistCoronavirus |
| #chinazi | #CoronavirusChino | #kungfuvirus | #chinaliedandpeopledied |
| #Chinavirus | #wuhanquarantine | #wuhanpnuemonia |  |
| #Wuhancoronavirus | #chinaIsAsshoe | #madeInChinaInfected |  |
| #wuhanpneumonia | #SanctionChina | #SingaporeVirus |  |
