# Supplementary figures and images for: Development of a COVID-19–Related Anti-Asian Tweet Data Set: Quantitative Study
Source: JMIR Form Res. 2023 Feb 28;7:e40403. doi: 10.2196/40403 (PMC9976773; doi:10.2196/40403)

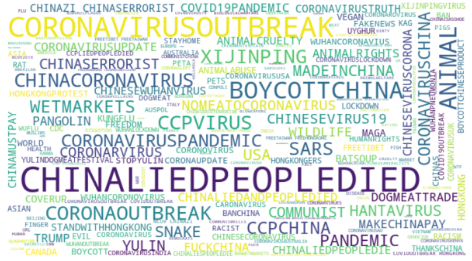

Supplement: Multimedia Appendix 2 [file formative_v7i1e40403_app2.png]
